# Foundation model embeddings for quantitative tumor imaging biomarkers

**DOI:** 10.21203/rs.3.rs-6630446/v1

**Published:** 2025-05-29

**Authors:** Hugo Aerts, Suraj Pai, Ibrahim Hadzic, Andrey Fedorov, Raymond Mak

**Affiliations:** Mass General Brigham ∣ Harvard Medical School; Mass General Brigham ∣ Harvard Medical School; Mass General Brigham ∣ Harvard Medical School; Brigham and Women's Hospital; Mass General Brigham and Harvard Medical School

## Abstract

Foundation models are increasingly used in medical imaging, yet their ability to extract reliable quantitative radiographic phenotypes of cancer across diverse clinical contexts lacks systematic evaluation. Here, we introduce TumorImagingBench, a curated benchmark comprising six public datasets (3,244 scans) with varied oncological endpoints. We evaluate ten medical imaging foundation models, representing diverse architectures and pre-training strategies developed between 2020 and 2025, assessing their performance in deriving deep learning-based radiographic phenotypes. Our analysis extends beyond endpoint prediction performance and compares robustness to common sources of variability and saliency-based interpretability. We additionally compare the mutual similarity of learned embedding representations across each of the models. This comparative benchmarking reveals performance disparities among models and provides critical insights to guide the selection of optimal foundation models for specific quantitative imaging tasks. We publicly release all code, curated datasets, and benchmark results to foster reproducible research and future developments in quantitative cancer imaging.

## INTRODUCTION

Precision oncology aims to revolutionize cancer care by tailoring treatments to the individual characteristics of each patient's tumor^1^. Central to this paradigm is the ability to characterize tumor biology, heterogeneity, and the tumor microenvironment, often non-invasively, to guide diagnosis, predict prognosis, and monitor therapeutic response^2^. Medical imaging modalities, including Computed Tomography (CT), Magnetic Resonance Imaging (MRI), and Positron Emission Tomography (PET), provide rich, spatially-resolved information about tissue structure and function, serving as key technologies in clinical oncology^3^.

Over the past decade, the field of quantitative imaging analysis, particularly radiomics, has emerged as a powerful tool to unlock deeper insights from these medical images beyond qualitative visual assessment^3-6^. Radiomics involves the extraction of a large number of quantitative features from medical images, converting them into mineable data that can potentially capture phenotypic characteristics related to underlying pathology. These features, when integrated with clinical and genomic data, have shown promise in predicting clinical endpoints such as diagnosis, patient survival, tumor recurrence, and treatment response across various cancer types^7^. However, traditional mathematical and statistical radiomics approaches face challenges related to feature reproducibility, standardization across different imaging parameters and scanners, and the inherent complexity of selecting and interpreting informative features from a high-dimensional space^8-10^.

The field of artificial intelligence, specifically deep learning, has witnessed transformative advancements, especially the development of Foundation Models (FMs) ^11^. These models, typically pre-trained on large and diverse datasets using self-supervised or unsupervised learning objectives, learn powerful and generalizable representations that can be adapted to various downstream tasks with minimal task-specific fine-tuning. Initially demonstrating remarkable success in natural language processing and computer vision ^12-15^, FMs are increasingly being explored within the medical domain^16-18^. In radiology, FMs have shown potential in tasks such as image segmentation, disease detection, report generation, and visual question answering^19,20^.

The capacity of FMs to implicitly learn complex features directly from image data presents an alternative to the handcrafted feature engineering inherent in traditional radiomics. By leveraging large-scale pre-training, FMs can potentially capture more robust and informative image representations, overcoming some limitations of conventional methods and advancing quantitative radiomics for precision oncology^21^. However, the proliferation of several FM architectures and pre-training strategies poses a significant challenge for researchers: selecting the most appropriate model for a specific quantitative radiomics task. While several benchmarks comparing FMs exist for general tasks^22-24^ and certain medical applications like report generation or segmentation^25-28^, a critical gap remains. There is currently no comprehensive, systematic benchmark specifically evaluating the performance and characteristics of different FMs as representation extractors for quantitative radiomics endpoints (i.e., diagnosis and prognosis prediction) across multiple anatomies and clinical cohorts. This lack of systematic comparison hinders informed model selection and reliable translation of FMs into radiomics research and practice.

To address this gap, we present the first comprehensive benchmark evaluating ten distinct, publicly available, pre-trained 3D foundation models for quantitative radiomics analysis. We assess their representational power across six diverse clinical cohorts spanning lung, kidney, and liver anatomies, on both diagnostic and prognostic prediction tasks. Our comparative analysis aims to present the relative strengths of these FMs in quantifying radiological phenotypes relevant to oncological outcomes. Our findings reveal that model performance is task- and dataset-dependent, with no single model universally superior, although certain models like FMCIB^21^, ModelsGenesis^29^, and VISTA3D^30^ demonstrate consistently strong performance across the evaluated scenarios. Beyond predictive performance, we investigate the robustness of the learned representations through test-retest reliability and input stability analyses, associate representations with salient image regions to gain insights into model interpretability, and explore the similarities between different FM representation spaces using representation alignment techniques. Furthermore, we introduce a unified, extensible software framework designed to facilitate the benchmarking of existing and future FMs on new tumor imaging datasets, thereby promoting standardized evaluation and accelerating the adoption of these powerful models within the quantitative imaging community.

## RESULTS

**Figure 1:**
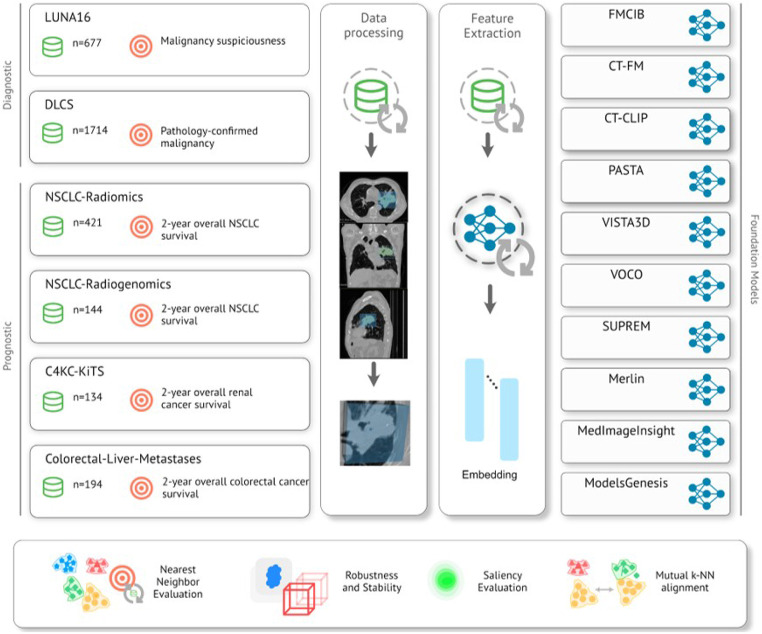
An overview of the data processing and analysis pipeline for TumorImagingBench. The pipeline includes multiple stages, including data processing with diverse datasets (LUNA16, DLCS, NSCLC-Radiomics, NSCLC-Radiogenomics, C4KC-KiTS, Colorectal-Liver-Metastases), feature extraction, feature evaluation through embedding and nearest neighbor evaluation, and target prediction. The right panel lists foundation models utilized for predictions, such as VISTA3D, Merlin, CT-CLIP, and others.

In this study, we established a comprehensive framework to compare ten distinct foundation models using six publicly available datasets. We assessed a variety of models that differed in architecture (convolutional vs. transformer-based), pre-training strategies (contrastive, supervised, generative, etc.), and data utilization (low-dose CT, all CT, CT + MRI, CT + MRI + US + others). These datasets address various endpoints across cancer types located in the lung, liver, and kidney. Foundation model embeddings were extracted from each dataset, with k-nearest neighbor models leveraging neighbor voting to predict endpoints. Figure 1 provides an overview of the datasets, models, and overarching framework.

### Diagnostic Performance of Foundation Models

For lung nodule malignancy diagnosis, performance varied considerably. On the LUNA16^31^ dataset, FMCIB demonstrated the highest diagnostic capability with an Area Under the Curve (AUC) of 0.886 (95% Confidence Interval [CI]: 0.871-0.9). ModelsGenesis ranked second with an AUC of 0.806 (95% CI: 0.795–0.816). Voco^32^ performed the worst, near random chance, with an AUC of 0.493 (95% CI: 0.468–0.519). VISTA3D achieved an AUC of 0.711 (95% CI: 0.692–0.730), while the remaining models yielded AUCs between 0.5 and 0.7 (Fig. 1 a). A similar ranking pattern occurred on the DLCS^33^ dataset, although with lower overall AUCs. FMCIB again led with an AUC of 0.675 (95% CI: 0.655–0.696), followed by ModelsGenesis at 0.645 (95% CI: 0.624–0.666). Voco (AUC: 0.507) and CTClip^19^ (AUC: 0.494) performed close to random chance. VISTA3D achieved an AUC of 0.607 (95% CI: 0.589–0.625), with other models scoring between 0.5 and 0.6 (Fig. 1a).

### Prognostic Performance Across Lung, Kidney, and Liver Cancer Datasets

In prognostic tasks, model performance generally decreased compared to diagnostics. For 2-year overall survival prediction in the NSCLC-Radiomics^34^ dataset, VISTA3D (AUC: 0.582, 95% CI: 0.545–0.62), FMCIB (AUC: 0.577, 95% CI: 0.549–0.605), and ModelsGenesis (AUC: 0.577, 95% CI: 0.539–0.614) were the top performers. CTClip (AUC: 0.449) and Voco (AUC: 0.526) performed worst (Fig. 1 a). On the NSCLC-Radiogenomics^35^ dataset for predicting NSCLC survival, VISTA3D (AUC: 0.622, 95% CI: 0.566–0.677) and CTFM^36^ (AUC: 0.620, 95% CI: 0.572–0.668) achieved the highest AUCs, followed closely by Merlin^37^ (AUC: 0.612) and ModelsGenesis (AUC: 0.609). Voco (AUC: 0.461) and CTClip (AUC: 0.510) showed the lowest performance (Fig. 1 a). For renal cancer prognosis (2-year overall survival) using C4KC-KiTS^38^, ModelsGenesis yielded the highest AUC of 0.733 (95% CI: 0.670–0.796), with SUPREM^39^ second at 0.718 (95% CI: 0.672–0.764). CTFM (AUC: 0.463) and CTClip (AUC: 0.493) performed worst (Fig. 1a). In predicting colorectal cancer liver metastases survival (Colorectal-Liver-Metastases^40^), only FMCIB achieved an AUC substantially above random chance at 0.572 (95% CI: 0.509–0.644). ModelsGenesis was the next best with an AUC of 0.530 (95% CI: 0.458–0.601), while other models performed near the random baseline (Fig. 1 a).

### Aggregate Performance and Ranking Across Datasets

Cross-dataset analysis revealed consistent performance patterns. FMCIB demonstrated strong overall performance, ranking first in three of the six datasets (LUNA16, DLCS, Colorectal-Liver-Metastases) and third in NSCLC-Radiomics (Fig. 1 b). ModelsGenesis also showed high consistency, ranking first or second in four datasets (LUNA16, DLCS, NSCLC-Radiomics, C4KC-KiTS) (Fig. 1b). Performance generally followed a trajectory starting highest in LUNA16, decreasing through DLCS and NSCLC prognostic tasks, partially recovering in C4KC-KiTS, and declining again in Colorectal-Liver-Metastases (Fig. 1c). Task-specific strengths were observed; FMCIB excelled in diagnostic tasks, while VISTA3D showed relatively stronger performance in prognostic tasks, achieving the top rank in NSCLC-Radiogenomics. The top three models (FMCIB, ModelsGenesis, VISTA3D) exhibited similar performance trends across the datasets (Fig. 1 c).

### Embedding Stability and Robustness to Input Variations

**Figure 2:**
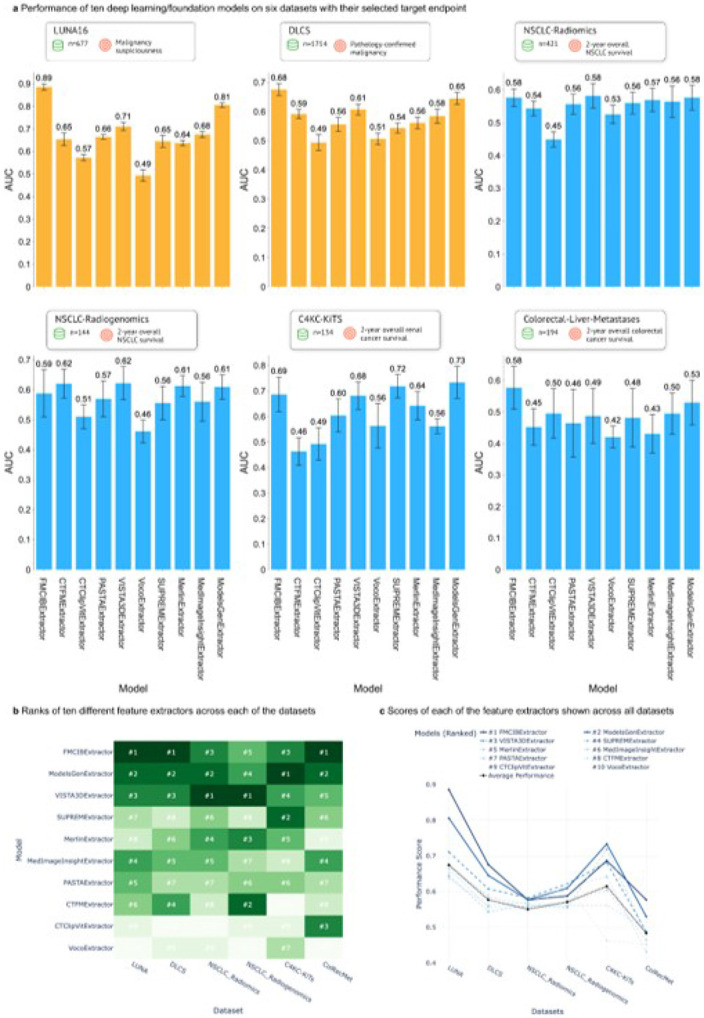
Comparative performance of deep learning feature extractors across cancer imaging datasets. **a.** AUC scores of nine models evaluated on six cancer datasets of TumorImagingBench with sample sizes noted **b.**Heat map showing model rankings (#1-#7) across datasets. **c.** Performance trajectories of top-ranked models across all datasets.

Test-retest stability evaluated on the RIDER dataset^41^, simulating scanning variability, was high for most models, showing average cosine similarities between 0.97 and 1.00. However, Merlin showed lower stability with an average similarity of 0.81, and CTClip scored 0.93 (Fig. 2a). Robustness to variations in input seed points (annotation noise) was assessed using Cohen's Kappa for agreement across 50 trials. CTFM showed the highest agreement (Kappa: 0.90), followed by SUPREM (0.87) and MedImageInsight^42^ (0.85). FMCIB maintained good agreement (Kappa: 0.70). In contrast, Voco demonstrated very poor agreement (Kappa: 0.05), with CTClip (0.29) and Merlin (0.36) also showing low robustness (Fig. 2b).

### Saliency Map Analysis for Explainability

Saliency maps generated via feature-based occlusion sensitivity^36^ indicated model focus areas. FMCIB and ModelsGenesis consistently produced saliency maps highlighting tumor regions across multiple datasets. VISTA3D maps sometimes focused on high-intensity bone structures when present, and a general tendency towards high-intensity regions was present across several models. CTClip, Voco, and Merlin failed to generate saliency maps clearly indicating tumor-specific regions (Fig. 2c).

### Similarity Relationships Between Foundation Model Embeddings

**Figure 3:**
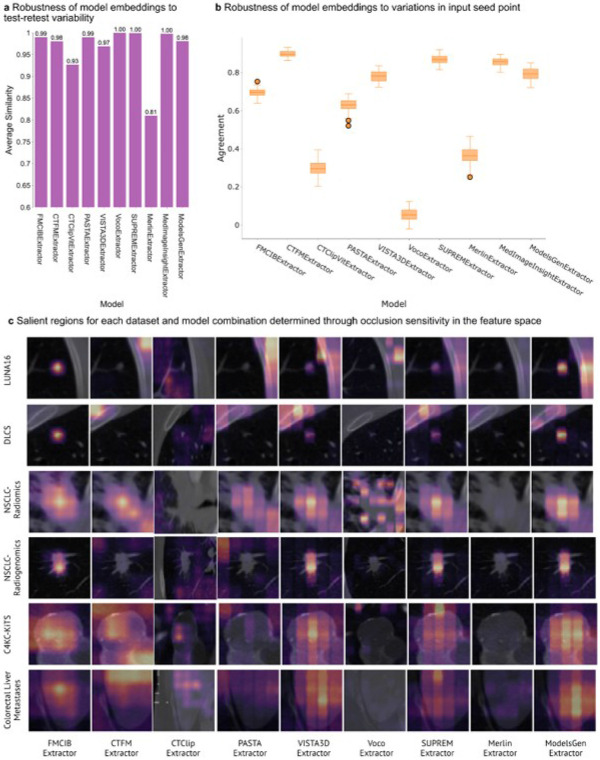
Robustness analysis and feature attribution of deep learning models across cancer imaging datasets. **a,** Average similarity scores demonstrating model embedding stability in test-retest scenarios, with most models showing high reproducibility (>0.95). **b,** Box plots displaying model agreement when varying input seed points, revealing significant variability in robustness across different extractors. **c,** Heatmaps of salient regions identified through occlusion sensitivity analysis across all model-dataset combinations, illustrating differences in feature attention patterns among the nine extractors across six cancer imaging datasets.

The relationships between the feature/embedding spaces learned by different models were examined using mutual k-nearest neighbor^43^ overlaps within each dataset. Consistent trends showed higher overlaps between embeddings from ModelsGenesis-VISTA3D, FMCIB-ModelsGenesis, and FMCIB-VISTA3D pairs across multiple datasets (Fig. 3a). These pairs frequently involved the top-performing models identified earlier. Alignment calculations further quantified these relationships; VISTA3D and ModelsGenesis embeddings showed the highest average alignment with FMCIB embeddings (scores of 2.85 and 2.69, respectively) (Fig. 3b). The maximum average alignment observed was between VISTA3D and ModelsGenesis (score of 3.89) (Fig. 3c, 3d), suggesting a convergence of feature representations among these high-performing models.

## DISCUSSION

In this study, we conducted the first comprehensive benchmarking of ten distinct foundation models (FMs) as feature extractors for quantitative tumor imaging tasks across six diverse, publicly available oncology datasets. Our evaluation includes predictive performance for diagnostic and prognostic endpoints, robustness to common sources of image variability, attribution analysis via saliency mapping, and an exploration of representational similarities between models. The results reveal significant differences in the ability of different FM embeddings for these downstream tasks, highlighting the challenges of model selection. Notably, we identified FMCIB, ModelsGenesis, and VISTA3D as exhibiting consistently strong performance across the range of datasets and clinical endpoints evaluated. Furthermore, this superior performance is often correlated with enhanced robustness and, for FMCIB and ModelsGenesis, biologically plausible saliency maps, suggesting these models capture more reliable and potentially interpretable quantitative phenotypes.

The popularity of FMs, pre-trained using varied architectures, datasets, and self-supervised objectives, presents both opportunity^21^ and complexity^16^ for domain-specific applications like quantitative tumor imaging. While numerous benchmarks exist for general computer vision^44-46^ and natural language processing^47,48^, and more recently within the broader medical field (e.g., BenchMD^49^, MedArena^25^), a dedicated evaluation framework comparing FMs specifically for their ability to generate predictive quantitative radiomic signatures remains missing. Previous benchmarking efforts in tumor imaging have primarily focused on standardizing traditional feature extraction pipelines or comparing classical machine learning algorithms applied to those features^50,51^. Our work directly addresses this gap, providing a much-needed resource for researchers seeking to leverage the representational power of FMs for biomarker discovery in oncology, analogous to how earlier benchmarks guided progress in other fields.

Our analysis across models spanning different eras of deep learning architectures (from UNets^52^ to Transformers^53^) and pre-training paradigms (restorative, contrastive, generative, segmentation-based) yielded several interesting observations. Perhaps most striking was the robust performance of ModelsGenesis, a relatively older model based on a simple UNet architecture and pre-trained using a restorative objective on only ~ 600 CT scans from a single LIDC dataset. Its consistent effectiveness across diverse anatomies and tasks suggests that its pre-training, focused on recovering corrupted inputs, may instill representations particularly robust to noise and adept at capturing fundamental tissue characteristics relevant for radiomics, even without exposure to vast datasets or explicit oncological tasks during pre-training. Similarly, FMCIB, which employs contrastive learning to achieve invariance against various image transformations^21^, also demonstrated strong, robust performance, reinforcing the hypothesis that pre-training objectives emphasizing robustness to transformations can yield powerful general-purpose embeddings for quantitative analysis. In contrast, VISTA3D^30^, leveraging segmentation pre-training on a large, diverse dataset including tumors, likely benefits from learning spatial features relevant to tumor characterization through its unique fine-grained supervoxel segmentation pre-training. The more recent PASTA^54^ model, despite sophisticated multi-stage pre-training involving synthetic tumors, yielded less performant embeddings in our benchmark, suggesting that while potentially powerful for fine-tuning on specific tasks (as shown in its original publication), its raw embeddings may not generalize as effectively for the broad range of quantitative radiomic tasks studied here. These findings collectively highlight that the choice of pre-training strategy and data significantly impacts the downstream utility of FM embeddings, and that newer or larger models are not invariably superior for every application.

As access to large-scale datasets and computational resources continues to grow, the landscape of FMs will undoubtedly become even more crowded. Comparing these models effectively, especially as performance on specific tasks begins to saturate, becomes increasingly critical. Inspired by concepts such as the Platonic Representation Hypothesis^43^, which suggests that optimal representations might converge towards a shared underlying structure, we investigated the similarity between the representation spaces of the FMs. Our analysis revealed that the models demonstrating stronger aggregate performance (notably FMCIB and ModelsGenesis) also exhibited significantly higher representational similarity, as measured by mutual neighbor analysis. This convergence among top-performing models, further corroborated by qualitative similarities in their saliency maps, suggests they may be learning overlapping, potentially fundamental, image features crucial for oncological prediction. This observation highlights possibilities regarding model interchangeability or ensembling strategies in the future, where understanding representational alignment could guide the selection of complementary models.

We acknowledge several limitations inherent in this study. First, our benchmark relies exclusively on six fully public datasets. While this choice maximizes accessibility and reproducibility, it limits the scale and diversity compared to potentially available restricted-access datasets. Second, our evaluation is currently image-only, potentially being unfair to the assessment of multimodal FMs that integrate text or other data types. However, the lack of availability of suitable public datasets with paired imaging and clinical text presents a significant challenge in this context. Third, our methodology focuses solely on evaluating the fixed embeddings extracted from the FMs, without task-specific fine-tuning. While fine-tuning can often boost performance on a target task, we argue that evaluating the raw embeddings provides a better assessment of the foundational representational power learned during pre-training – a strong foundational embedding should inherently capture rich image properties beneficial for downstream tasks, and should correlate with fine-tuning performance. Future work could explicitly compare embedding performance versus fine-tuning outcomes within this framework. Finally, for downstream prediction, we employed a simple k-nearest neighbor classifier to minimize confounding factors from complex modeling choices, though exploring a wider range of classifiers could be future work..

In conclusion, this study introduces a comprehensive benchmark for evaluating foundation models in quantitative tumor imaging, providing critical insights into the performance, robustness, and underlying representational characteristics of ten prominent models across diverse oncological tasks. Our findings reveal that model selection requires careful consideration, with FMCIB, ModelsGenesis, and VISTA3D emerging as particularly promising candidates. Crucially, we present not only these results but also an open-source, extensible framework for systematic and reproducible integration of new foundation models and dataset. We hope this work will significantly push forward the adoption and rigorous evaluation of these foundation models, accelerating progress in quantitative imaging for precision oncology.

## METHODS

### Design of TumorImagingBench Evaluation

The TumorImagingBench is constructed to evaluate tumor imaging (radiomic) signatures' capacity to quantify diverse radiological phenotypes of cancer. It encompasses six publicly available datasets: LUNA16 and DLCS for lung cancer diagnostics, focusing on nodule malignancy; NSCLC-Radiomics and NSCLC-Radiogenomics for assessing prognosis post-surgery and/or radiotherapy in non-small cell lung cancer patients; C4KC-KiTs for renal carcinoma prognosis post-partial or radical nephrectomy; and Colorectal-Liver-Metastases for assessing prognosis in colorectal cancer patients with liver metastases post-hepatic resection. The selection of diverse endpoints across these datasets highlights the generalizability of selected radiomics signatures.

#### LUNA16^31^:

A curated selection from the LIDC-IDRI database, featuring 888 thoracic CT scans (diagnostic and lung screening) from 7 academic centers and 8 imaging companies. It includes 1,186 lung nodules, each annotated for location and attributes like internal composition, calcification, and malignancy by a consensus of at least 3 out of 4 radiologists. For a specific evaluation mentioned, a subset of 677 nodules was chosen, all having at least one indication of malignancy suspicion.

#### DLCS^55^:

A dataset from the Duke Health system featuring 2,487 nodules from 1,613 patients. Nodules, initially flagged by AI and verified by a medical student (with selective radiologist oversight), include 3D bounding boxes, Lung-RADS annotations, and cancer outcomes. The selection followed Lung-RADS v2022 criteria, focusing on nodules ≥ 4 mm or in central/segmental airways. A subset of the dataset containing 1714 scans made publicly available with pathology confirmed malignancies was used in this study.

#### NSCLC-Radiomics^34^:

An independent test set for prognostication networks derived from the MAASTRO Clinic (Maastricht, NL). This set consisted of CT scans from 421 patients, selected from a cohort of 422 individuals with stage I-IIIB NSCLC treated with radiation therapy. Key characteristics include annotated primary Gross Tumor Volumes (GTVs), delineated by radiation oncologists using FDG PET-CT scans (Siemens Biograph, +/− contrast), and patients being right-censored for two-year survival. The chosen end-point for prediction is 2-year survival from treatment date. .

#### NSCLC-Radiogenomics^35^:

A dataset of 211 stage I-IV NSCLC patients from Stanford University and the Palo Alto VA (recruited 2008-2012, referred for surgery) who had preoperative CT and PET/CT scans (variable equipment/protocols). Tumor segmentations reviewed by two radiologists are available for 144 patients. The dataset also includes molecular data (EGFR/KRAS/ALK mutations, gene expression, RNA-seq). Our study focused on 133 patients with annotated Gross Tumor Volumes (GTVs), right-censored for two-year survival. This subset served as an independent test set for prognostication and subsequent biological investigation of our networks. The chosen end-point for prediction is 2-year survival post surgery.

#### C4KC-KiTS^38^:

A dataset of patients who underwent partial or radical nephrectomy for renal tumors at the University of Minnesota Medical Center between 2010 and 2018 were considered for inclusion in our analysis. Cases lacking preoperative arterial phase abdominal CT imaging were excluded. From the eligible population, 300 cases were randomly selected for potential inclusion. Of these, 210 had complete tumor segmentation data available. After excluding patients lost to follow-up prior to the event of interest, our final cohort for this study comprised 134 patients. For multi-tumor cases, we used the largest tumor volume to determine the seed point for our computational analysis. The chosen end-point for prediction is 2-year survival post nephrectomy.

#### Colorectal-Liver-Metastases^40^:

A dataset of single-institution consecutive series of patients who underwent colorectal liver metastases (CRLM) resection with matched preoperative CT scans. Inclusion required: pathologically confirmed CRLM, available pathologic data of non-tumoral liver parenchyma and tumor, and preoperative portal venous contrast-enhanced MDCT within 6 weeks of resection. Patients with 90-day mortality, <24 months follow-up, preoperative hepatic artery infusion chemotherapy, local tumor ablation, >3 wedge resections, or no visible tumor on preoperative imaging, were excluded. For our analysis, we selected the largest tumor from each patient, resulting in a final cohort of 194 patients after excluding those lost to follow-up. The chosen end-point for prediction is 2-year survival post resection..

### Selection of FM Radiomic Embedding Models

We selected ten pre-trained models as embeddings to establish a radiomic signature for provided computed tomography images. Models span from 2020 to the latest in 2025, capturing prevalent design choices in pre-trained model development over five years. The earliest model, ModelsGenesis, employs a simple UNet convolutional network, while the most recent model, PASTA, utilizes a sophisticated nnUNet framework in a multimodal scheme. CT-ViT and MedImageInsight, recent models, incorporate joint text-vision approaches and transformer architectures. Table 1 presents a comparative analysis of these approaches based on several critical design choices.

**FMCIB** is a foundation model designed to distinguish between lesions and non-lesions at the patch level in medical imaging. It aims to enhance the detection and characterization of cancerous lesions by leveraging self-supervised learning techniques.

**CT-FM** is a large-scale 3D image-based pre-trained model specifically developed for various radiological tasks, including segmentation and classification. It was trained on a substantial dataset of CT scans and demonstrated superior performance across multiple tasks compared to state-of-the-art models. The model's architecture allows it to effectively cluster anatomical regions and identify similar structures across scans, making it a robust tool for medical image analysis

**CT-CLIP** is a novel 3D adaptation of the CLIP model, designed for multi-abnormality detection in chest CT scans. It utilizes contrastive learning to align CT volumes with corresponding radiology reports, enabling zero-shot classification capabilities. This model excels in detecting multiple abnormalities without the need for extensive manual annotations, showcasing its potential for efficient clinical applications

**PASTA** is a 3D-CT foundation model that addresses data scarcity in oncology by synthesizing lesions across various organs and tumor types. It utilizes a generative model to create a large dataset of synthetic CT scans, which enhances its training for lesion segmentation and vision-language alignment tasks. PASTA has shown exceptional performance in cross-domain transfer learning, outperforming existing models in multiple evaluation tasks

**VISTA3D** is a versatile imaging segmentation and annotation model that supports both automatic and interactive segmentation of 3D medical images. It is the first unified foundation model to achieve state-of-the-art performance across 127 classes and is designed to facilitate efficient human correction through its interactive features. VISTA3D integrates a novel supervoxel method to enhance zero-shot performance, making it a significant advancement in 3D medical imaging

**VOCO** is a large-scale 3D medical image pre-training framework that leverages geometric context priors to learn consistent semantic representations. It is built on a substantial dataset of CT volumes and employs a novel pretext task for contextual position predictions. VOCO has demonstrated superior performance across various downstream tasks, establishing itself as a leading model in the field of medical imaging.

**SUPREM** is a suite of pre-trained models that provides state-of-the-art performance in organ and tumor segmentation tasks. It is based on supervised pre-training methodologies and has been shown to outperform models trained from scratch. SUPREM's architecture includes various backbones, allowing it to adapt effectively to different medical imaging tasks

**Merlin** is a vision-language foundation model designed for interpreting abdominal CT scans. It integrates structured electronic health record (EHR) data and unstructured radiology reports to perform a variety of tasks, including zero-shot classification and report generation. Merlin's architecture allows it to generalize across multiple downstream tasks, making it a versatile tool for clinical applications

**MedImageInsight** is a lightweight foundation model for medical imaging that spans multiple modalities, including X-ray, CT, and MRI and is trained via image-text/label/age alignment. It achieves state-of-the-art performance on various datasets and supports both classification and image retrieval tasks. The model is designed to be adaptable and efficient, making it suitable for real-world clinical applications.

**ModelsGenesis** is a collection of models built from unlabeled 3D imaging data using a restorative reconstruction-based self-supervised method. It aims to generate powerful application-specific target models through transfer learning. The models demonstrate strong performance across various medical imaging tasks, emphasizing their potential for broad applicability in clinical settings

### Embedding/Feature extraction configuration

Our feature extraction configuration involved a very similar procedure across all the models. For the segmentation focused models, we took the embeddings from the last layers of the encoder and added an average pooling on top to compress the feature representations in the spatial dimension. For non-segmentation models, the last layer of the model was taken in a similar fashion and average pooled. For MedImageInsight, a 2D model, we averaged across all 3D slices to obtain our embedding, as recommended in the original study.

### K-Nearest Neighbor Modelling setup

Final layer embeddings were used as radiomic features for each sample. These features were used to predict outcomes using a k-nearest neighbor model. We also used cosine distance for the neighbor model. Models were trained using 10-fold cross-validation and results were aggregated across the 10 runs. Optuna hyperparameter tuning was used for each run independently to select the optimal number of neighbors for the task from a range of 1 to 50. 95% confidence intervals were calculated using the 10-fold cross-validation.

### Robustness and Saliency Evaluation

We evaluated model robustness through multiple complementary approaches. First, to assess test-retest reliability, we utilized the RIDER dataset–a collection of chest CT scans from 26 patients where each patient underwent two scans within a 15-minute interval. For each model, we calculated the cosine similarity between embeddings generated from these paired scans. Higher cosine similarity values indicate greater robustness to normal scanning variability. To evaluate sensitivity to input variations, we simulated annotation variability by generating 50 random perturbations of each seed point. These perturbations followed a three-dimensional multivariate normal distribution (zero mean, diagonal covariance matrix) with a variance of 16 voxels in each dimension. We then trained models on one trial and compared predictions across all trials, measuring agreement using Cohen's Kappa after converting continuous predictions to categorical values. We implemented occlusion sensitivity analysis to identify image regions most influential to model predictions. This approach systematically occludes different regions of the input image and measures resulting changes in the output embeddings using cosine distance. Regions causing larger embedding deviations when occluded are considered more salient to the model's feature extraction process. We generated and compared these saliency maps across all models to assess their focus areas.

### Mutual K-Nearest Neighbor Evaluation

To compare feature representations between models, we employed mutual k-nearest neighbor analysis. For each sample, we identified its 10 nearest neighbors in the embedding space generated by each model. We then quantified the overlap between neighbor sets across different models. This overlap metric reveals similarities in how different models structure their feature spaces and cluster similar samples, providing insight into which models learn comparable representations despite architectural differences.

## Figures and Tables

**Figure 4:**
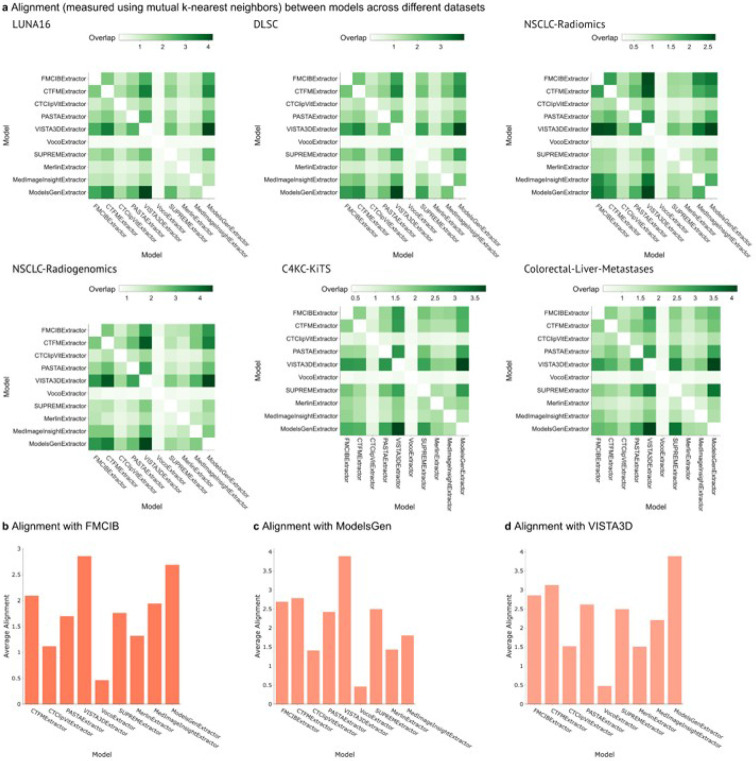
Alignment and average alignment between models. **a.** Heatmaps illustrating model overlap across different datasets, measured using mutual k-nearest neighbors, shown in varying shades of green to indicate the degree of overlap. (**b-d**) Bar charts presenting average alignment for top performing models: FMCIB, ModelsGen, and VISTA3D, highlighting alignment differences across models.

**Table 1:** Comparative analysis pre-trained/foundation models with their corresponding pre-training frameworks, architectural specifications, and training datasets. The table highlights various models, their meta-architectural designs, and structural configurations. Pre-training datasets are summarized with relevant characteristics. The specific downstream evaluation tasks that utilized these pre-trained models are also enumerated to demonstrate model applicability and performance across domains.

Model	Meta-architecture / Pre-training design	Architecture	Dataset	Params	Evaluated Tasks
FMCIB^21^	Tumor positive + Negative− mining SimCLR	3D ResNet50	11,467 scans from DeepLesion	184M	Patch-based diagnosis and prognosis
CT-FM^36^	3D image-based contrastive pre-training promoting awareness of 3D structure.	3D SegResNet	148k scans from Imaging Data Commons (multiple datasets)	77M	Whole-body and tumor segmentation, head CT triage, medical image retrieval, semantic understanding
CT-CLIP^19^	Contrastive language-image pretraining framework for 3D chest CTs using radiology reports	3D ViT	25,692 scans from CT-RATE dataset	25M	Multi-abnormality detection, case retrieval, zero-shot classification
PASTA^54^	Two-stage process focusing on semantic segmentation and text-image alignment on synthetic tumors	nnUNet	30k scans from PASTA-GEN30k dataset	127M	Few-shot and zero-shot segmentation, tumor staging and prognosis,lesion report generation
VISTA3D^30^	Supervised multi-instance training along with supervoxel supervision and separate heads for interactive segmentation	3D SegResNet	11454 scans from 15 different datasets	175M	Segmentation tasks across various anatomical structures and lesion (+ Interactive)
VOCO^32^	Large-scale 3D medical image pre-training with geometric context priors.	3D SwinUNETR	160k CTs from 30 public datasets	295M	Segmentation, classification, registration, and vision-language tasks
SUPREM^39^	Supervised pre-training on AbdomenAtlas 1.1, combining large-scale datasets with per-voxel annotations.	3D UNet	9,262 scans from AbdomenAtlasl.1	19M	Segmentation tasks across multiple datasets, demonstrating transfer learning capabilities
Merlin^37^	Vision-language foundation model for 3D CT, trained with EHR and radiology reports	3D UNet	15,331 CT scans along with radiology reports	270M	Zero-shot classification, phenotype classification, radiology report generation, 3D segmentation
MedImageInsight^42^	Two-tower architecture optimized with UniCL objective f.	2D ViT	3.7M image-text/label/age pairs across several datasets	616M	Image-text search, image-image search, report generation, and task fine-tuning
ModelsGenesis^29^	Image restoration on 3D Chest CT volumes	3D UNet	623 scans from LUNA16 dataset	7M	Segmentation and classification tasks across five target 3D applications

## Data Availability

All datasets used in this study are publicly available and can be downloaded from their respective Zenodo, IDC^56^ and TCIA^57^ sources. Links to the dataset are available through the data citations.

## CODE AVAILABILITY

The complete pipeline used in this study can be accessed either from the AIM webpage or directly on https://github.com/AIM-Harvard/TumorImagingBench.

Code included for 1) Data preprocessing and creating annotation files, 2) Extracting features through a systematic interface for each model across all dataset, 3) Evaluation pipelines for building models and post-evaluation.
